# Supplementary material for: High-Sensitivity Troponin T Testing: Consequences on Daily Clinical Practice and Effects on Diagnosis of Myocardial Infarction
Source: J Clin Med. 2020 Mar 12;9(3):775. doi: 10.3390/jcm9030775 (PMC7141275; doi:10.3390/jcm9030775)
Supplement: Supplementary file 1 [file jcm-09-00775-s001.pdf]

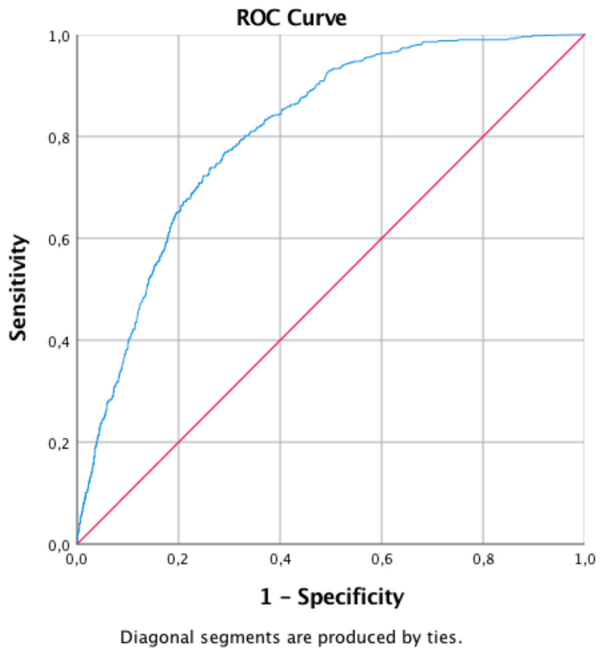


**Figure S1.** ROC Curve for hs-TnT for prediction of in-hospital mortality, AUC = 0.81.

ROC: receiver operating characteristic; AUC: area under the curve; hs-TnT: high-sensitivity troponin T.
